# Immunotherapy in triple negative breast cancer: beyond checkpoint inhibitors

**DOI:** 10.1038/s41523-022-00486-y

**Published:** 2022-11-09

**Authors:** Yara Abdou, Atta Goudarzi, Jia Xin Yu, Samik Upadhaya, Benjamin Vincent, Lisa A. Carey

**Affiliations:** 1 Lineberger Comprehensive Cancer Center, University of North Carolina, Chapel Hill, NC 27599 USA; 2 Department of Medicine, University at Buffalo, Buffalo, NY 14203 USA; 3 Parker Institute for Cancer Immunotherapy, San Francisco, CA 94129 USA; 4 Cancer Research Institute, New York, NY 10006 USA

**Keywords:** Breast cancer, Cancer immunotherapy

## Abstract

The development of immunotherapy agents has revolutionized the field of oncology. The only FDA-approved immunotherapeutic approach in breast cancer consists of immune checkpoint inhibitors, yet several novel immune-modulatory strategies are being actively studied and appear promising. Innovative immunotherapeutic strategies are urgently needed in triple negative breast cancer (TNBC), a subtype of breast cancer known for its poor prognosis and its resistance to conventional treatments. TNBC is more primed to respond to immunotherapy given the presence of more tumor infiltrating lymphocytes, higher PD-L1 expression, and higher tumor mutation burden relative to the other breast cancer subtypes, and therefore, immuno-oncology represents a key area of promise for TNBC research. The aim of this review is to highlight current data and ongoing efforts to establish the safety and efficacy of immunotherapeutic approaches beyond checkpoint inhibitors in TNBC.

## Introduction

Triple negative breast cancer (TNBC) accounts for ~15–20% of incident breast cancers and is characterized by negativity for estrogen receptor (ER), progesterone receptor (PR), and human epidermal growth factor receptor 2 (HER2). TNBC encompasses more than one molecular subtype, with the majority of tumors found to be of the basal‐like RNA expression phenotype. However, other molecular subtypes can be present; including the HER2-Enriched group, Luminal A, Luminal B, Claudin‐low, and a few normal‐like molecular subtypes^1^. More recent studies have further refined TNBC subtyping by identifying targetable molecules highly expressed in each TNBC subtype for effective immune-based treatment strategies^2^. Despite significant biological heterogeneity within this disease, and recent advances in neoadjuvant and adjuvant therapy, overall, TNBC has been associated with a higher risk for recurrence and disease progression, and poorer outcomes. Once metastatic, this breast cancer subtype has an estimated median overall survival (OS) of 16 months and a median progression-free survival (PFS) of 5.6 months with standard chemotherapy in the first-line setting^3^. There is an unmet clinical need to develop more efficacious and less toxic therapies for patients with TNBC.

The development of immunotherapy agents has revolutionized the field of oncology with durable responses and improvements in OS^4^. Different tumor types including melanoma, renal cell carcinoma, lung cancer, and bladder cancer have greatly benefited from immune checkpoint inhibitors (ICIs)^5^, however, in breast cancer, early trials with ICI as monotherapy achieved limited objective responses^6^. Immunotherapy for treatment of breast cancer has not been prioritized, largely because breast cancer has been considered poorly immunogenic making it less likely to respond to immunotherapies^7–9^. Nevertheless, there is increasing evidence to suggest the presence of variable immunogenic activity in different breast cancer subtypes^10,11^, with TNBC likely exhibiting the strongest immunogenicity^12^. TNBC has been shown to have a higher proportion of tumor infiltrating lymphocytes (TILs)^13–15^ compared to other subtypes, relatively high tumor mutational burden^16^ and PD-L1 expression^17^, and survival associations with degree of T cell and B cell infiltration^18^, making immunotherapy a promising option against this disease. This has encouraged the development of more immunotherapy drugs to treat TNBC patients.

The first FDA accelerated approval of an ICI for the treatment of breast cancer came in March 2019 when the anti-PD-L1 antibody atezolizumab was approved in combination with nab-paclitaxel as a first-line treatment for patients with PD-L1-positive, metastatic TNBC based on the IMpassion130 trial^19^. Continued approval of this combination was contingent upon results of the IMpassion131 trial evaluating first-line atezolizumab and paclitaxel in TNBC, however, updated results in 2021 indicated that the trial failed to meet the primary end point of PFS superiority in the frontline treatment of patients with PD-L1 positivity and there was no difference in survival advantage in the PD-L1–positive nor the intention to treat population^20^. Based on this data, atezolizumab-chemotherapy combination has been withdrawn as an indication for treatment of TNBC. Alternatively, KEYNOTE-355 continues to demonstrate a clinically meaningful improvement in PFS with pembrolizumab, an anti-PD-1 antibody, in combination with chemotherapy, vs. chemotherapy alone among patients with PDL-1 positive, metastatic TNBC with CPS ≥ 10^3,21^. Based on these results, on November 2020, the FDA granted accelerated approval to pembrolizumab in combination with chemotherapy for the treatment of patients with metastatic TNBC whose tumors express PD-L1 (CPS ≥ 10). Similarly, in July 2021, pembrolizumab was approved for high-risk, early-stage, TNBC in combination with chemotherapy as neoadjuvant treatment and continued as adjuvant treatment, based on results from the KEYNOTE-522 trial showing substantial benefit in terms of event-free survival and distant recurrence-free survival, regardless of PD-L1 status^22^. There are several additional ongoing clinical trials evaluating the role of other types of immunotherapy combinations in TNBC^23–25^. Ongoing efforts have revolved around modulating the tumor microenvironment (TME) to increase breast cancer immunogenicity and the therapeutic efficacy of immunotherapeutic agents. The objective of this review is to discuss emerging immunotherapy agents in TNBC patients, highlighting therapies beyond ICIs.

## Landscape of TNBC immunotherapies

**Fig. 1:**
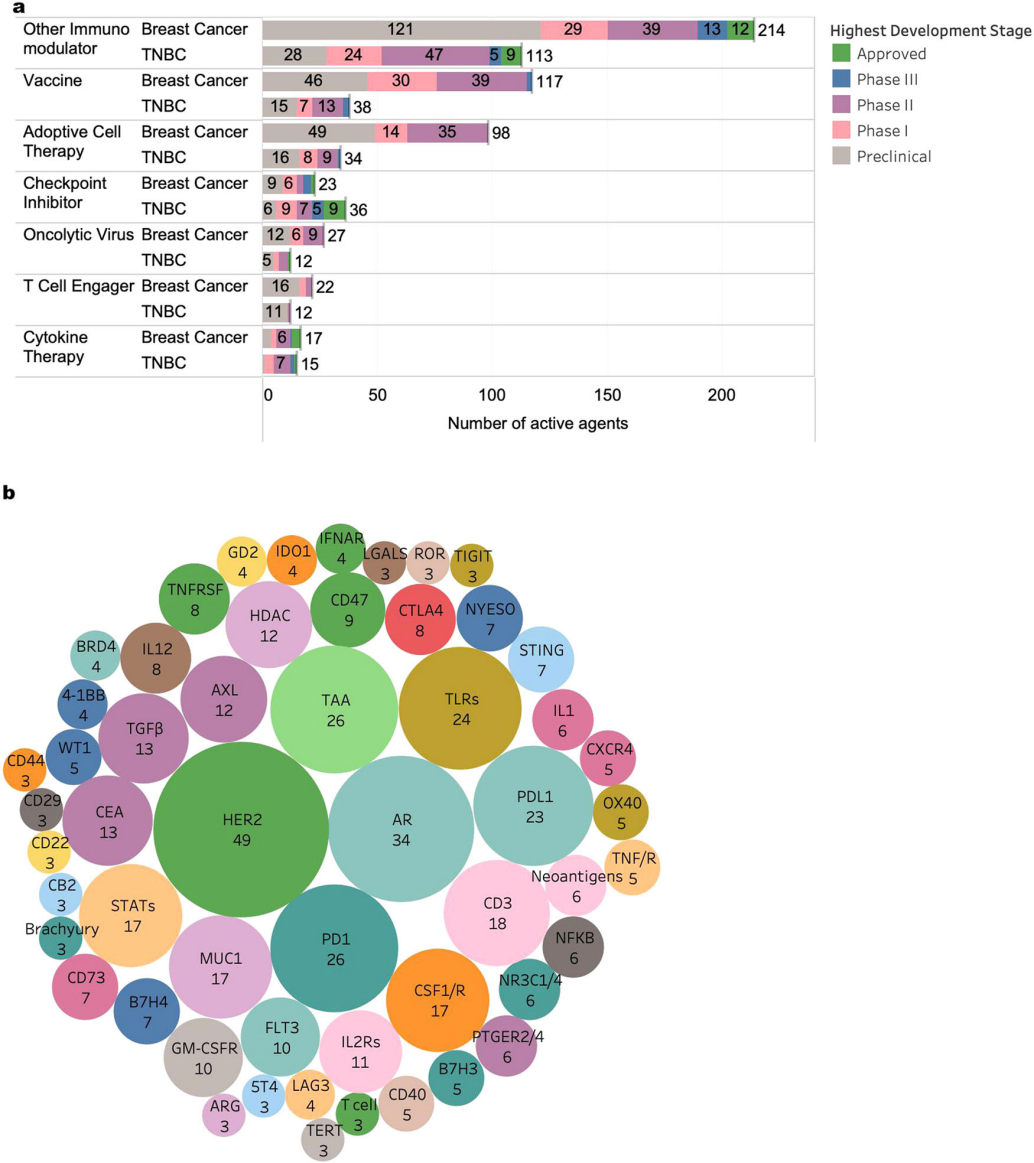
Immuno-oncology (IO) agents in development for breast cancer and triple-negative breast cancer (TNBC). **a** Number of IO agents in active development that are being explored in breast cancer and TNBC. “Checkpoint inhibitor” category includes only PD-1, PD-L1, and CTLA-4 targeted agents, while other immunomodulators that fall outside of “vaccine”, “adoptive cell therapy”, “oncolytic virus”, “cytokine therapy”, and “T cell engagers” are grouped into “other immunomodulators”. These include immunomodulators to natural killer cells, B cells, and other immune cells. **b** Most common targets of IO agents explored in breast cancer, where only targets with at least three agents in active development are shown. TAA tumor-associated antigens.

Using their immuno-oncology (IO) database, the Cancer Research Institute (CRI) explored the number and type of IO agents being developed for use in breast cancer, specifically TNBC (Fig. 1a)^26^. The data pull completed in March 2022 showed 778 total agents actively being developed for use in breast cancer and TNBC, at various developmental stages. ICIs, which have had recent approvals in TNBC, lag behind cancer vaccines, adoptive cell therapies and “Other Immunomodulators”; these include immunomodulators to natural killer (NK) cells, B cells and other immune cell agonists or antagonists. Newer modalities such as T cell engagers, which are bispecific antibodies that simultaneously bind with the T-cell and the tumor cell, are mostly in preclinical or early phase, while cytokine-based therapies, have progressed to later phases of development given its approved use in other cancers such as melanoma, particularly peginterferon alfa-2b and interleukin-2. Due to the antigen-specific nature of some of these IO agents, CRI further explored the most common targets of these IO agents (Fig. 1b). While HER2 is the most common target identified in breast cancer, other major targets include, adenosine receptor targets, nonspecific tumor-associated antigens (TAA), immune checkpoints (PD-1, PD-L1), toll-like receptors (TLRs), and colony-stimulating factor-1 (CSF1/R).

**Fig. 2:**
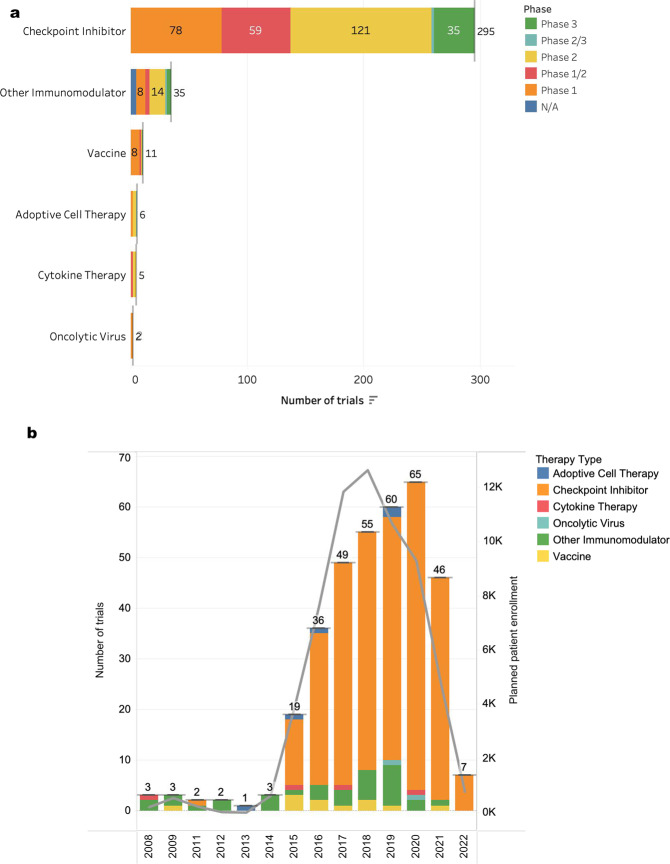
Clinical trials for triple-negative breast cancer using IO agents as of March 2022 data pull from clinicaltrials.gov database. **a** Number of clinical trials at various study phases across different types of IO therapy. **b** Number of trials using various IO therapies from 2008 to present (*incomplete 2022 trials due to data pull date). Line graph represents patient enrollment for each indicated year.

Clinical trial data was pulled from clinicaltrials.gov as of March 2022 to select for TNBC trials utilizing at least one IO agent, regardless of recruitment status. Despite the development of many categories of IO agents, ICIs dominate in TNBC, representing 295 of 354 (83%) of TNBC IO trials (Fig. 2a). Most of the IO studies are still in early phases, with ~40 trials currently in phase 3. Many of these studies have yet to disclose efficacy. We further divided the trials based on initiation date to estimate how many trials could possibly have efficacy data in the near term (Fig. 2b). The majority of trials were initiated in the past 6 years, peaking in 2020, with a noted decline in the last 2 years, presumably due to the COVID-19 pandemic. As these trials complete, we expect more immunotherapy options for TNBC patients to emerge beyond checkpoint inhibitors.

## Vaccine therapy

Therapeutic vaccines typically target known breast tumor antigens to enhance tumor-specific immune responses through active immunization. Some newer methods generate vaccines based on the mutanome of a patient’s particular tumor^27^. Vaccines can variably generate cytotoxic CD8^+^ T-cell (CTLs) and NK responses, as well as affect tumor growth directly by altering the TME through chemokines^28^. Different modalities of therapeutic vaccines exist utilizing either peptides, carbohydrates, DNA or RNA (usually recombinant), whole cells, or dendritic cells (DC)^28^. Peptides and carbohydrates present limited epitopes, whereas genetic vaccines can generate the entirety of epitopes associated with an antigen^29^. DC vaccines utilize the antigen-presenting and T-cell stimulating properties of DC, as well as modulating cytokines and chemokines to control inflammation and lymphocyte migration to help produce long-lasting anti-tumor effects^30^.

### Peptide and carbohydrate-based vaccines

Monovalent peptide and carbohydrate-based vaccines generate an immune response to a single antigen. Sialyl-Tn (STn) is a carbohydrate antigen associated with poor prognosis in multiple cancers including breast cancer^31^. In a phase III study, patients receiving endocrine therapy plus a vaccine consisting of STn conjugated to the carrier protein keyhole-limpet hemocyanin (KLH) showed increased OS in 180 metastatic breast cancer patients (all subtypes) compared to immunization with just the KLH protein (37 vs. 31 months). The analysis did not take into consideration the molecular subtypes, and the number of TNBC patients included in the study was not disclosed, therefore there is unknown applicability to TNBC^32^.

AE37 is the Ii-Key hybrid of the Major Histocompatibility Complex (MHC) class II peptide that is capable of stimulating CD4^+^ helper T cells. The final analysis of the randomized phase II trial of AE37 + GM-CSF vaccine verses GM-CSF alone for the prevention of breast cancer recurrence in node-positive or high-risk, node negative patients, showed no significant differences in the 5-year disease-free survival (DFS) between treatment arms. However, the subgroup analysis showed that the TNBC cohort (*n* = 50) had an ~35% reduction in the relative risk of recurrence^33,34^. Further studies are needed to evaluate the clinical benefit of this vaccine in TNBC.

Folate receptor alpha (FRα) is another target for a therapeutic vaccine in patients with breast cancer. A phase 1 study of FRα peptide vaccine in breast and ovarian cancer patients was well tolerated and elicited augmented immunity in more than 90% of patients examined, with responses that persisted at least 12 months^35^. Two phase II trials are investigating cyclophosphamide combined with FRα peptide vaccine in large cohorts of women with TNBC (NCT03012100, NCT02593227).

Vaccines utilizing the membrane-bound carbohydrate antigens NeuGCGM3 and Muc1, have shown trends toward better clinical outcome and prolonged periods of no evidence of disease (NED) respectively, in advanced-stage assorted breast cancer^36–38^. Uncontrolled studies of vaccines targeting telomerase (hTERT) and survivin, an anti-apoptosis protein, have produced periods of stable disease and immunological response in studies of advanced-stage breast cancers of undisclosed subtypes^39,40^. Other peptide-based vaccines including P10s (a peptide that mimics a carbohydrate antigen) and personalized peptide vaccine (PPV) (where vaccine antigens are selected based on pre-existing immunoglobulin G (IgG) responses) have shown enhanced immunogenicity and possible clinical benefits in early clinical studies, especially in TNBC subgroups^41,42^.

### Polyvalent peptide vaccines

Polyvalent peptide vaccines target multiple antigens in a single vaccine. Takahashi and colleagues reported 9 clinical responses among 79 breast cancer patients using peptides selected from a pool of 31 peptides targeting various antigens based on HLA typing and in vivo antibody response, including 1 TNBC with complete response and a second TNBC with partial response^42^. Additionally, 9 of 10 TNBC patients in this study showed augmented antibody responses, and 7 of 14 patients showed augmented cytotoxic cellular response^42^. Berinstein and colleagues used a 7-peptide vaccine targeting 7 different tumor antigens in a cohort of 23 adenocarcinomas that included three metastatic breast cancers and showed SD lasting 8 months in one patient and CD8^+^ T-cell responses in all three patients^43^. Studies using PVX-410, a four-peptide vaccine targeting three antigens, and Galinpepimut, a four-peptide vaccine targeting WT1, have shown clinical effects in studies of smoldering myeloma and mesothelioma, respectively^44,45^. Clinical trials are currently underway for TNBC patients utilizing PVX-410, and Galinpepimut vaccines (Table 1).

**Table 1:** Clinical trials for breast cancer treatment using vaccine therapy in TNBC.

Vaccine	Patients	Phase	NCT	Status
Peptide—AE37 with Pembrolizumab	Stage IV TNBC	II	NCT04024800	Active, not recruiting
Peptide—P10s-PADRE with or without standard chemotherapy	Stage II–III TNBC	II	NCT02938442	Recruiting
Neo-antigen peptide—long peptide with nab-paclitaxel and Durvalumab	Stage IV TNBC	II	NCT03606967	Recruiting
Multi-peptide—PVX-410 with Durvalumab	Stage II–III TNBC	Ib	NCT02826434	Active, not recruiting
Multi-peptide—PVX-410 with or without Pembrolizumab	Stage IV or inoperable HLA A2+ TNBC	Ib	NCT03362060	Active, not recruiting
Multi-peptide—Galinpepimut (WT1)	Select advanced cancers including TNBC	I/II	NCT03761914	Active, not recruiting
Multi-peptide—Folate Receptor Alpha with GM-CSF following cyclophosphamide	Stage Ib–IV TNBC	II	NCT03012100	Recruiting
DNA—adenovirus CEA, MUC1, and brachyury Peptide—RAS, CEA, and brachyury; with various chemotherapeutics and targeted inhibitors	Progressive TNBC post standard therapy	Ib/II	NCT03387085	Active, not recruiting
DNA—vaccinia p53	Solid tumors that failed prior therapy (including TNBC)	I	NCT02432963	Active, not recruiting
Neo-antigen DNA—with or without Durvalumab	Stage II/III TNBC	I	NCT03199040	Active, not recruiting
Neo-antigen RNA liposomes	All stage TNBC	I	NCT02316457	Active, not recruiting
DC—Autologous DC’s pulsed with autologous neo-antigen peptides	All stage TNBC	I	NCT04105582	Completed as of June 2022
DC targeted against Her2/Her3 with pembrolizumab and a cytokine modulation regimen	Stage IV TNBC or HER2+ breast cancer	II	NCT04348747	Recruiting

### Neoantigen vaccines

Neoantigen vaccines use peptides that are unique to particular mutations in the patient’s tumor and not present in normal cells, therefore theoretically avoid host self-tolerance^27,46^. These vaccines were shown to elicit robust anti-tumor immune responses through activation of tumor antigen-specific CD8^+^ and CD4^+^ T cells^47,48^. A randomized phase II study of nab*-*paclitaxel, durvalumab and neoantigen vaccine vs. nab-paclitaxel and durvalumab alone in metastatic TNBC is currently recruiting (NCT03606967)^49^. Another phase I clinical trial of a neoantigen vaccine with or without durvalumab to treat stage II–III TNBC patients who have residual disease after neoadjuvant therapy is also recruiting (NCT03199040).

### Genetic vaccines

Genetic vaccines utilize recombinant DNA of an antigen in a vector of isolated plasmid, virus, bacterial or yeast cell. The antigens are then expressed by host cells (in the case of isolated plasmid), or are expressed by the viral, bacterial or yeast cells in the vaccines. These methods are advantageous because these vectors utilize complete target complementary DNA (cDNA) sequences and therefore can generate multiple antigenic epitopes per target. Furthermore, some vectors themselves are immunogenic, which potentiates the targeted immune response, and the vectors can further be transfected with cDNA of T-cell stimulating proteins to enhance that effect. The PANVAC vaccine is a poxvirus transfected with cDNA of CEA and MUC-1, as well as the T-cell stimulating proteins B7.1, ICAM-1 and LFA-3^50^. This vaccine has produced clinical responses in 3 patients, including 1 TNBC^51,52^. Four vaccines—PANVAC; a related CEA vaccine with the same stimulating proteins, a yeast-based vaccine targeting brachyury; a transcription factor associated with tumor epithelial-mesenchymal transition, and viral vaccines containing the NY-ESO-1 antigen, have all produced SD in varying proportions of carcinomas, although these studies contained small numbers of breast cancer patients and did not have control groups^52–55^. A study of INVAC-1, an isolated plasmid vaccine containing cDNA of hTERT, in a population of 26 adenocarcinomas, including 5 patients with stage III-IV TNBC, showed two instances of PR and 15 cases of SD of up to 10 months^56^. Additionally, a vaccine of P53-transfected vaccinia cells along with pembrolizumab produced regression of cutaneous metastasis in a woman with stage IV TNBC^57,58^. Clinical trials of genetic vaccines containing CEA1, MUC1, and Brachyury, and P53 are currently underway for TNBC patients (Table 1).

### Dendritic cell vaccines

DC vaccines use autologous patient-derived DC as a vaccine vector; either loaded/transfected with tumor antigens or fused with autologous or allogenic preparations. A study of ten patients with stage III TNBC investigated the use of neoadjuvant DC loaded with WT1 and cyclin B1 antigens, administered with neoadjuvant chemotherapy, showing 5 cases of pathological complete response (pCR)^59^. Svane and colleagues showed that, of 32 breast cancer patients of mixed subtypes, 11 had disease stabilization using an autologous DC vaccine pulsed with multiple p53 peptides. 8 of these patients had increased CD8+ T-cell responses^60^. DC vaccines fused with autologous tumor cells demonstrated significantly longer than expected 3-year PFS amongst 66 ER-/PR- patients^61^, and 2 cases of PR among 10 patients with stage IV breast cancer^62^. Other studies of DC vaccine with folate receptor alpha (FRα)^63^ and hTERT^64^ peptides have shown T-cell activation in early clinical studies. In a phase II randomized study of 275 patients in the adjuvant setting, a DC vaccine targeting the E75 peptide derived from the HER2 protein was tested with trastuzumab in breast cancer patients with low HER2 expression^65^. Although there was no DFS benefit overall, subgroup analysis showed a significant improvement in DFS in the TNBC cohort: 86.6% vs. 70.6% in the placebo group at 3 years post-treatment. Three early phase clinical trials of DC vaccines in TNBC are currently in early stages, one using DC vaccine loaded with neo-antigen peptide, a second using DC loaded with WT1 and cyclin B1 antigens, and a third using DC vaccine targeting HER2/HER3 antigens with pembrolizumab in breast cancer patients with brain metastasis (Table 1).

## Adoptive cell therapies

T cells play an important role in cell-mediated immunity. Chimeric antigen receptor T-cell (CAR-T) therapy and T-cell receptor (TCR)–engineered T-cell therapy are two types of adoptive cell therapies (ACTs) that can genetically modify the patient’s natural T cells ex vivo and inject them back into the patient’s body to make them tumor-specific and enhance their ability to destroy tumor cells^66^. The mechanisms by which they recognize antigens are quite different^67^. CAR T-cells are engineered to recognize only surface antigens through its antigen-binding site of antibody fragments fused to the T-cell. A major advantage of CAR-T cells is relatively high antigen-binding affinity, typically in the nanomolar range compared to micromolar TCR binding affinities. On the other hand, TCRs use an alpha-beta chain heterodimer to recognize intracellular antigens that are expressed on the cell surface by MHC. Therefore, TCRs may have an advantage over CAR-T in solid tumors, given they are able to target a wider range of antigens^67,68^.

### Chimeric antigen receptor-modified T (CAR-T) cell therapy

Current CAR T-cell therapies are approved for the treatment of certain patients with non-Hodgkin lymphomas and leukemias. Despite the exciting clinical efficacy seen in hematologic malignancies, several challenges still exist for the use of CAR T-cell therapies in solid tumors. Many studies are focusing on overcoming these challenges and improving the efficacy of this novel approach to immunotherapy in solid tumors^69^. A key challenge for CAR-T therapy to overcome in breast cancer is improving CAR-T cell infiltration into tumors, which may be overcome by using potent stimulation of antigen-presenting cells to make chemotactic cytokines combined with administration of CAR-T cells^70^.

A cell-surface molecule, c-Met, was found to be highly expressed in ~50% of breast tumors, and is associated with basal-like TNBC, supporting the production of a CAR-T cell specific for c-Met^71^. Tchou and colleagues published results from a phase 0 trial (NCT01837602) evaluating the safety and feasibility of intratumoral injections of RNA c-Met-CAR-T cells in patients with c-Met–expressing metastatic breast cancer^72^. Four out of six patients had TNBC. Results showed that the c-Met-CAR-T-cell injections were well tolerated and elicited an inflammatory response intratumorally. A phase 1 trial (NCT03060356) to evaluate the feasibility, safety and efficacy of intravenously administered mRNA c-Met-CAR-T cells in patients with metastatic breast cancer is underway.

Mesothelin expression was found to be highly expressed in TNBC and is associated with poor prognosis^73^. This prompted the production of mesothelin-specific CAR-T cells which have shown to have in vitro anti-tumor cytotoxicity against primary breast tumor cells^74^. Preliminary results from the phase I/II study (NCT02414269) evaluating the safety and efficacy of mesothelin-targeted CAR-T cells in patients with advanced solid tumors showed evidence of CAR-T cell anti-tumor activity and no major toxicities^75^. A phase I clinical trial (NCT02792114) to evaluate the safety and tolerability of mesothelin-targeted CAR-T cells in patients with pretreated metastatic mesothelin-expressing breast cancer is currently recruiting.

Mucin 1 (MUC1) is a heterodimeric protein that is highly expressed in over 90% of TNBC and is associated with poor prognosis^76,77^. Tumor MUC1 specific CAR-T cells were shown to have potent anti-tumor cytotoxicity both in vitro and in vivo^78^. A Phase I study of anti-MUC1 CAR-T cells for patients with advanced MUC1 positive breast cancer is currently recruiting (NCT04020575). Receptor tyrosine kinase-like orphan receptor 1 (ROR1) is expressed on tumor cells of primary breast cancer and high expression of ROR1 has been associated with poor prognosis^79^. ROR1 CAR-T cells were shown to confer a potent anti-tumor effect against TNBC cell lines in vitro^80^. A phase I study of ROR1 CAR-T cells in patients with advanced ROR1+ malignancies including TNBC is currently ongoing (NCT02706392). Preliminary results from 6 patients (4 with TNBC) showed no dose-limiting toxicities with some evidence of disease control^81^. NKR-2 are autologous T cells genetically engineered to express a receptor normally present on natural killer (NK) cells called NK group 2D (NKG2D). THINK (THerapeutic Immunotherapy with NKR-2) is a multinational Phase I study (NCT03018405) that is currently ongoing, to assess the safety and clinical activity of multiple administrations of autologous NKR-2 cells in seven refractory cancers, including TNBC.

### T cell receptor gene therapy

NY-ESO-1 is an antigen found to be overexpressed in TNBC and demonstrated high immunogenicity in this subgroup of patients^82^. An early phase study (NCT01967823) of anti-NY ESO-1 T cell receptor (TCR) gene therapy in advanced solid tumors expressing NY-ESO-1 is underway. Melanoma antigen family A (MAGE-A) antigen identifies an aggressive subgroup of TNBC that may benefit from immune response augmentation^83,84^. A Phase II clinical trial utilizing T cell receptor immunity targeting MAGE-A3 for patients with metastatic cancer, including breast cancer, is currently recruiting (NCT02111850). Carcinoembryonic antigen (CEA) is a known tumor marker of breast cancer and has been associated with negative prognostic factors^85^. A phase I trial of anti-CEA T Cells is assessing safety and optimal dosing for this therapy in metastatic breast cancer patients (NCT00673829). Kita-Kyushu Lung Cancer Antigen-1 (KK-LC-1) or CXorf61 is frequently expressed by several epithelial cancers including TNBC^86^. KK-LC-1 TCR gene-engineered T cells showed evidence of specific recognition of KK-LC-1 positive TNBC tumor cell lines in vitro and mediated regression of KK-LC-1 positive tumors in vivo^87^. Future clinical testing of KK-LC-1 directed T cell therapy in TNBC is warranted.

## Tumor infiltrating lymphocytes

Autologous TILs are emerging as a new type of immunotherapy in breast cancer. TILs are a group of lymphocytes infiltrating the tumor’s stroma and actively engage in tumor destruction. A recent case report was of a patient with chemo refractory hormone receptor–positive metastatic breast cancer who had a durable complete response after adoptive transfer of neoantigen-specific TILs^88^. A phase II study to evaluate the efficacy of autologous TIL therapy (LN-145) as a single therapy in metastatic TNBC patients is currently recruiting (NCT04111510).

## Oncolytic virus therapy

Oncolytic viruses have shown promising therapeutic efficacy in preclinical breast cancer models. Currently, different types of oncolytic viruses are being developed to target early and metastatic breast cancer. TVEC is a genetically modified type 1 herpes simplex virus (HSV-1) to preferentially replicate in tumor cells^89^. An early-phase trial including nine patients with TNBC found that adding the oncolytic virus talimogene laherparepvec (T-VEC) to standard chemotherapy in the neoadjuvant setting was well tolerated and showed promising efficacy^90^. Although the sample size was small, results showed increases in cytotoxic T cell infiltration in most of the resected tumor specimens in addition to a reduction in regulatory T cells^90^. Results of the phase II trial (NCT02779855), with forty patients enrolled, confirmed that the addition of TVEC to neoadjuvant chemotherapy was safe with increased rates of pathological complete response^91^. Another phase II trial evaluated the efficacy of intratumoral T-VEC as monotherapy for inoperable locoregional recurrence of breast cancer with or without distant recurrence (NCT02658812). The study showed that intratumoral T-VEC as monotherapy did not have optimal outcomes due to uncontrolled disease progression, and administration of concurrent systemic therapy may be warranted^92^. Furthermore, an early phase, multi-institutional study to evaluate the safety of intrahepatic T-VEC injections in combination with atezolizumab in TNBC patients with liver metastasis (NCT03256344) observed no dose-limiting toxicities, and reported one patient that had a partial response^93^. A phase II trial of in situ oncolytic virus therapy consisting of adenovirus-mediated expression of herpes simplex virus thymidine kinase plus ganciclovir and stereotactic body radiation therapy followed by pembrolizumab was tested in twenty-eight patients (eighteen with PD-L1 negative tumors) with locally advanced or metastatic TNBC (NCT03004183). Results showed that the combination was well-tolerated, with promising efficacy in heavily pretreated metastatic TNBC patients. One patient had complete response, and has remained disease-free without any systemic therapy for 39 months despite early discontinuation of pembrolizumab due to Grade 3 pneumonitis^94^.

LTX-315, a novel oncolytic peptide, has shown promising results when administered as monotherapy or in combination with pembrolizumab in TNBC patients with transdermally accessible tumors (NCT01986426)^95^. In a TNBC in vivo model, LTX-315 combined with doxorubicin induced immune-mediated changes in the TME and demonstrated promising therapeutic potential^96^.

Pelareorep, a serotype 3 reovirus, was evaluated in patients with metastatic breast cancer, including TNBC. The final analysis of the randomized phase II study showed that pelareorep was well tolerated and the combination arm had a significantly longer OS^97^. The AWARE-1 trial (NCT04102618) is currently enrolling early-stage breast cancer patients to 5 different cohorts with pelareorep. Six TNBC patients will be treated with combination of pelareorep and atezolizumab. Preliminary data demonstrated enhanced inflammatory markers in the tumor after the combination treatment^98^.

Adenovirus is the most studied oncolytic virus platform in breast cancer research. A phase I trial using adenovirus, ICOVIR-7, enrolled three patients with advanced breast cancer. While the drug was rendered to be safe, the breast cancer patients did not meet their efficacy endpoints^99^. On the other hand, an oncolytic adenovirus coding for GMCSF (Ad5/3-D24-GMCSF) was shown to induce anti-tumoral immunity and efficacy in patients with advanced breast tumors, including TNBC^100^.

Several other oncolytic viruses, including Maraba, Measles, Polio, Coxsackie, Vaccinia, Newcastle disease, have been tested in breast cancer in vivo and ex vivo models, paving the way for future safety studies in humans^101^. Ongoing clinical trials are currently investigating the efficacy of different oncolytic viruses in solid tumors and breast cancer (Table 2).

**Table 2:** Clinical trials for breast cancer treatment using oncolytic virotherapy approaches.

Virus	Adjunct therapy	Disease	Phase	NCT ID	Status
Measles (MV-NIS)	None	Metastatic Breast Cancer HNSCC	I	NCT01846091	Active, not recruiting
Talimogene Laherparepvec (HSV)	Paclitaxel	TNBC	I/II	NCT02779855	Active, not recruiting
Talimogene Laherparepvec(HSV)	Nivolumab Ipilimumab	Breast cancer (includes TNBC)	I	NCT04185311	Active, not recruiting
HSV-1 (ONCR-177)	± Pembrolizumab	Advanced/refractory solid tumors (includes breast cancer)	I	NCT04348916	Recruiting
Poliovirus (PVSRIPO)	None	TNBC	I	NCT03564782	Recruiting
Adenovirus (ADV/HSV-tk)	SBRT, Pembrolizumab Valacyclovir	TNBC NSCLC	II	NCT03004183	Active, not recruiting
Poxvirus (JX-594)	Cyclophosphamide	TNBC Sarcoma	I/II	NCT02630368	Recruiting
Poxvirus (Pexa-Vec)	Ipilimumab	Advanced solid tumors (includes TNBC)	I/II	NCT02977156	Active, not recruiting
Maraba virus (MG1MA3)	± Adenovirus	Advanced solid tumors (includes breast cancer)	I/II	NCT02285816	Active, not recruiting
Vaccinia virus (p53MVA)	Pembrolizumab	Refractory solid tumors (includes TNBC)	I	NCT02432963	Active, not recruiting
Vaccinia virus (TBio-6517)	Pembrolizumab	TNBC Solid tumors	I/II	NCT04301011	Recruiting
Reovirus (pelareorep)	Atezolizumab	Breast cancer (includes TNBC)	I	NCT04102618	Recruiting

## Cytokine gene therapy

Cytokines are soluble proteins that mediate cell-to-cell communication. There is evidence that cytokines play important roles in inflammatory and immune responses, making them a critical target for anti-tumor responses^102^. IL-2 and IFN-α are the only two cytokines that received Food and Drug Administration (FDA) approval for cancer treatment, however their use was limited by their high toxicity profile^103^. There is a renewed interest in exploring the anti-tumor properties of cytokine-based drugs, not only as monotherapy, but also in combination with other immunotherapy agents. IL-12 is one of the most potent anti-tumoral cytokines^104^. Prior studies have shown that intratumoral administration of a recombinant adenovirus encoding IL-12 (AdIL-12) resulted in significant tumor regression in breast cancer animal models^105^. The Phase 1 pilot study of IL-12 monotherapy in treatment refractory, metastatic TNBC patients showed evidence of treatment-related increase in CD8^+^ TIL density and enhanced antigen presentation after intratumoral administration of IL-12^106^. The Phase 2 KEYNOTE-890 trial (NCT03567720) evaluated the efficacy of intratumoral tavokinogene teleplasmid, a plasmid encoding IL-12, followed by electroporation and pembrolizumab in metastatic TNBC patients. Evidence of enhanced tumor immunogenicity was observed in addition to 28.6% objective responses, regardless of PDL-1 status^107^. NKTR-214, also known as bempegaldesleukin, is an engineered cytokine that specifically stimulates the IL-2 receptor. NKTR-214 has been tested in a phase 1 trial (NCT02869295) targeting metastatic solid tumor, including TNBC. Based on a favorable safety profile and evidence of a substantial increase in CD8^+^ T and NK cells within the TME after NKTR-214 treatment^108^, a phase I/II clinical trial combining NKTR-214 and nivolumab was initiated (NCT02983045). 38 patients with metastatic solid tumors were enrolled, preliminary results showed early evidence of clinical activity with an ORR of 13.2% and no dose-limiting toxicities^109^.

### Novel immunotherapy targeting the TME

Several clinical trials are investigating novel therapeutic approaches to overcome the immunosuppressive elements of the breast cancer TME. Some of these approaches include expanding effector T-cells, NK cells and other immunostimulatory cells while suppressing regulatory T cells, tumor-associated M2 macrophages and myeloid derived suppressor cells (MDSCs)^110^. The goal is to modify the TME by increasing anti-tumor immune responses, suppressing pro-tumor immune responses to produce high immunogenicity and ultimately, a more favorable response to cancer immunotherapy.

SD-101; an intratumoral toll-like receptor 9 (TLR9) agonist, is a novel immune priming strategy that was shown to modify the TME by increasing local production of type 1 Interferon, resulting in cytotoxic T-cell infiltration and an anti-tumor response^111^. The combination of neoadjuvant SD-101 and pembrolizumab in addition to weekly paclitaxel followed by doxorubicin and cyclophosphamide was investigated in a treatment arm of the I-SPY 2 trial (NCT01042379). Results showed a non-statistically significant increase in estimated pCR rates in seventy-five patients with high-risk, HER2-negative stage II/III breast cancer (~30 patients with TNBC)^112^.

Another novel and interesting method for TME remodeling and enhancing therapy outcomes is through targeting the chemokine system. Chemokines are small signaling proteins that direct the migration and trafficking of immune cells within the TME. Chemokines play a critical role in shaping the immune cell composition and mediating the balance between anti-tumor and pro-tumor responses^113^. CCL5 (ligand for CCR5) and CXCL9, CXCL10, CXCL11 (ligands for CXCR3) are the main chemokines attracting CTLs, type-1 helper (Th1) and NK cells producing an inflammatory response in the TME. On the other hand, production of CCL2, CCL22, and CXCL12 promotes intratumoral infiltration of suppressive immune cells, such as CXCR4+ MDSCs, M2 macrophages, and CCR4+/CXCR4+ regulatory T-cells (Tregs)^114–116^. A single arm study (NCT03599453) is investigating how well chemokine modulation therapy works when given prior to check point inhibitor (pembrolizumab) in patients with metastatic TNBC. The study uses a combination of celecoxib (COX-2 inhibitor), recombinant interferon alfa-2b (IFN-alpha), and rintatolimod (a selective toll-like receptors 3 (TLR-3) agonist) given systemically on 3 consecutive days, 1 week apart for a total of 2 weeks prior to initiating pembrolizumab every 3 weeks. Theodoraki and colleagues have shown that the combination of TLR-3 agonist with COX-2 blockers (and/or with IFN-alpha), allowed selective enhancement of type-1 immunity, promoting CTLs migration, while suppressing Treg/MDSC attraction^117^.

## Future directions

Immune checkpoint inhibition added to chemotherapy improves survival outcomes in TNBC patients. Several other novel immunotherapeutic approaches show promise in this patient cohort. While the IO field continues to grow, a deeper understanding of breast cancer and its microenvironment is still needed to overcome the apparent low immunogenicity in this disease and to optimize immune-therapeutic approaches to their full potential. At least three conditions are required to ensure optimal immunotherapy responses: (1) generation of tumor antigen-specific T cells, (2) influx of T cells into the tumor, (3) reversal of immunosuppression mechanisms operating in the tumor immune microenvironment. Though tumor immunotherapy has classically focused on the T cell arm of the adaptive immune system, other approaches, such as tumor antigen-specific B cells, may be important as well to immunotherapy response in breast cancer. Thus, it is likely that combination immunotherapy strategies will be needed going forward. With an increasing number of clinical trials and available immunotherapeutic agents, we anticipate that these promising strategies will improve clinical outcomes in TNBC patients, while decreasing our dependence on cytotoxic therapies.
